# Angiotensin receptor blockers might be protective against hepatic steatosis after liver transplantation

**DOI:** 10.1186/s12876-023-02781-9

**Published:** 2023-05-15

**Authors:** Ahad Eshraghian, Alireza Taghavi, Hamed Nikoupour, Saman Nikeghbalian, Seyed Ali Malek-Hosseini

**Affiliations:** 1 Shiraz Transplant Center, Abu-Ali Sina Hospital, 71994-67985 Shiraz, Iran; 2 Shiraz Transplant Center, Abu-Ali Sina Hospital, Shiraz University of Medical Sciences, Shiraz, Iran

**Keywords:** Angiotensin receptor blockers, Non-alcoholic fatty liver disease, Liver transplantation, Hypertension, Hepatic steatosis, Losartan

## Abstract

**Background:**

Hepatic steatosis is an increasing complication in liver transplant recipients. Currently, there is no pharmacologic therapy for treatment of hepatic steatosis after liver transplantation. The aim of this study was to determine the association between use of angiotensin receptor blockers (ARB) and hepatic steatosis in liver transplant recipients.

**Methods:**

We conducted a case-control analysis on data from Shiraz Liver Transplant Registry. Liver transplant recipients with and without hepatic steatosis were compared for risk factors including use of ARB.

**Results:**

A total of 103 liver transplant recipients were included in the study. Thirty five patients treated with ARB and 68 patients (66%) did not receive these medications. In univariate analysis, ARB use (P = 0.002), serum triglyceride (P = 0.006), weight after liver transplantation (P = 0.011) and etiology of liver disease (P = 0.008) were associated with hepatic steatosis after liver transplantation. In multivariate regression analysis, ARB use was associated with lower likelihood of hepatic steatosis in liver transplant recipients (OR = 0.303, 95% CI: 0.117–0.784; P = 0.014). Mean duration of ARB use (P = 0.024) and mean cumulative daily dose of ARB (P = 0.015) were significantly lower in patients with hepatic steatosis.

**Conclusion:**

Our study showed that ARB use was associated with reduced incidence of hepatic steatosis in liver transplant recipients.

## Introduction

Non-alcoholic fatty liver disease (NAFLD), recently suggested to rename as metabolic dysfunction associated fatty liver (MAFLD), is a rapidly growing disease worldwide and is going to become the most common cause of liver cirrhosis and liver transplantation [1, 2]. It has been suggested that the rising prevalence of diabetes mellitus and obesity are mainly responsible for the increasing prevalence of NAFLD [3]. De novo development or recurrence of hepatic steatosis have been frequently reported after liver transplantation. De novo steatosis or non-alcoholic steatohepatitis (NASH) have been reported in up to 33% of post liver transplant biopsies 3 years after transplantation [4]. Using ultrasound, we have previously reported that the occurrence of steatosis after liver transplantation was up to 56% in patients with NASH and more than 26% in patients with cryptogenic cirrhosis [5].

The main treatment of NAFLD is life style modification including exercise, weight loss and dietary regimen [6, 7]. It has been reported that 10% weight reduction in patients with NASH will result in improvement of not only steatosis but also inflammation and fibrosis [8]. On the other hand, several medications targeted against different pathogenic mechanisms of hepatic steatosis have been suggested and prescribed as treatment of NAFLD in clinical practice [9, 10]. At present, there is no suggested medication for treatment or prevention of NAFLD after liver transplantation and current recommendations are mainly derived from clinical evidences in non- transplant setting. Considering high rate of NAFLD after liver transplantation, that might negatively impact liver allograft, other preventive or therapeutic interventions might be required.

Angiotensin receptor blockers (ARB) are a group of agents that binds to angiotensin II type I receptor and result in inhibition of renin angiotensin system [11]. These are widely used for treatment of arterial hypertension, diabetic nephropathy and hypertensive cardiovascular disease [12]. While some ARB may rarely cause cholestatic hepatitis some recent studies have reported beneficial effects of inhibition of renin angiotensin system for the treatment of NAFLD/NASH [13, 14]. However, clinical application of this class of anti-hypertensive medications in liver transplant recipients has not been well elucidated. This study aimed to investigate association between use of ARB and NAFLD in liver transplant recipients.

## Materials and methods

We used data set of Shiraz Transplant Center, a referral liver transplant center in Iran. We used data of adult (> 18 years) liver transplant recipients including information about age, sex, underlying liver disease, date of transplantation, laboratory data, post-transplant diabetes mellitus (PTDM), hyperlipidemia, hypertension, rejection episodes, immunosuppression regimens and other medications. All the patients included in the study were followed until April 2019. Measurement of height and weight was performed and recorded at the time of last clinical visit. Body mass index (BMI) was calculated using this formula: Weight (kg)/ Height (m)^2^. Patients labeled as cryptogenic cirrhosis with BMI > 25 Kg/m^2^ were considered as having NASH as underlying cause of liver cirrhosis.

Hepatic steatosis after liver transplantation was diagnosed by abdominal ultrasonography. Patients were examined with both supine and left posterior oblique positions in longitudinal, transverse, and oblique scanning planes. Hepatic steatosis was diagnosed based on these 4 criteria in ultrasound: liver brightness, hepatorenal echo contrast, and vascular blurring, deep attenuation. Grade of liver steatosis was described based on the severity of liver echogenicity and classified as grade 0, grade 1, grade 2, and grade 3; grade 0: normal echogenicity; grade 1: slight, diffuse increase in fine echoes in liver parenchyma; grade 2: moderate impaired visualization of intrahepatic vessels and diaphragm with diffuse increase in fine echoes of liver parenchyma; grade 3: marked increase in the fine echoes of liver parenchyma [15].

Liver transplant recipients with hepatic steatosis after liver transplant diagnosed by ultrasound were compared to those without hepatic steatosis as controls. Patients and controls were matched based on the time of follow-up, sex, age, and time of liver transplant.

Liver transplant recipients were routinely visited at our outpatient clinic and ARB users and non-users were distinguished based on the prescription of ARB by clinicians and confirmation of ARB use by patients. The total time of ARB treatment had to be more than 3 months by the patient to be included in the study. Defined daily dose (DDD) was defined as the assumed average maintenance dose per day for ARB used for their main indication in adults. Cumulative defined daily dose (cDDD) of ARB was assessed in relation to duration and daily dose of use for losartan and valsartan. cDDD was calculated by summing the DDD from the beginning to the und of use of ARB in an individual patient and classified to < 20 and ≥ 20 gram [16]. Other ARB were not used by our patients.

### Statistical analysis

Continuous and categorical variables were compared using Student’s t-test and Chi-square test, respectively. Non-parametric Mann Whitney U test was used when appropriate. Numeric variables were presented as means ± standard deviation and categorical variables were presented as percents and counts. Variables with statistically significant difference during univariate analysis in patients with and without hepatic steatosis were included in a regression model. Logistic regression analysis was used to identify independent variables associated with NAFLD after liver transplantation. SPSS 18.0 (SPSS Inc.; Chicago, IL, USA) software was used for statistical analysis. A P- value of < 0.05 was considered statistically significant.

## Results

Totally, 103 liver transplant recipients were included in the study. Thirty five (34%) patients treated with ARB. They were compared to 68 patients (66%) who did not receive these medications. In ARB group, 27 patients (77.1%) received losartan and 8 patients (22.8%) received valsartan. All patients who received ARB had hypertension and 11 patients had diabetic albuminuria. No adverse reaction related to use of ARB was reported in our patients treating with these medications. Characteristics of patients with and without ARB therapy are summarized in Table 1. Baseline characteristics of patients with and without hepatic steatosis are outlined in Table 2. The 2 groups were not different in terms of matching variables including age, sex, and components of metabolic syndrome. Totally, 54 patients developed hepatic steatosis after liver transplantation. Mean time form liver transplant to development of hepatic steatosis was 18 ± 14.87 months. In univariate analysis, 11 (20.4%) patients with hepatic steatosis used ARB versus 24 (49%) patients without hepatic steatosis (OR: 0.266; 95% CI: 0.112–0.634; P = 0.002). Underlying cause of cirrhosis (NASH versus other causes) (OR: 2.928; 95% CI: 1.314–6.526; P = 0.008), serum triglyceride (P = 0.006) and weight after liver transplant (P = 0.011) were also associated with development of hepatic steatosis after liver transplantation (Table 2). In a regression model, we included serum triglyceride, ARB use, weight after liver transplantation, and etiology of liver disease. In regression model, ARB use was associated with lower likelihood of hepatic steatosis in liver transplant recipients (OR = 0.303, 95% CI: 0.117–0.784; P = 0.014) (Table 3).

**Table 1:** Characteristics of patients with and without angiotensin receptor blockers therapy

	ARB use	No ARB use	P-value
Age (year)	50.52 ± 11.17	51.45 ± 10.01	0.682
Sex (men/women)	21/14	44/24	0.771
Weight (kg)	72.47 ± 16.29	77.86 ± 15.77	0.110
Height (cm)	167.81 ± 11.70	168.30 ± 9.56	0.826
BMI (kg/m^2^)	25.62 ± 5.12	27.55 ± 6.15	0.119
PTDM	20	35	0.368
Hyperlipidemia	22	36	0.179
AST (IU/L)	23.12 ± 9.79	26.58 ± 12.80	0.183
ALT (IU/L)	29.15 ± 20.65	32.60 ± 20.58	0.443
ALK. Ph (IU/L)	221.84 ± 94.82	258.32 ± 121.92	0.142
T. Bilirubin (mg/dL)	0.98 ± 0.50	0.95 ± 0.49	0.807
FBS (mg/dL)	126.91 ± 54.98	141.96 ± 74.00	0.301
TG (mg/dL)	190.72 ± 118.59	207.88 ± 130.49	0.530
Cholesterol (mg/dL)	188.93 ± 52.64	186.84 ± 63.45	0.871
HDL (mg/dL)	46.09 ± 12.86	41.93 ± 14.12	0.162
LDL (mg/dL)	103.15 ± 36.36	98.61 ± 38.99	0.581
Hepatic steatosis	11	43	0.003

ARB: angiotensin receptor blockers; PTDM: Post transplant diabetes mellitus; BMI: Body mass index; AST: Aspartate aminotransferase; ALT: Alanine aminotransferase; ALK. Ph: Alkaline phosphatase; FBS: Fasting blood sugar; LDL: Low density lipoprotein; HDL: High density lipoprotein; TG: Triglyceride

**Table 2:** Characteristics of patients with and without hepatic steatosis after liver transplantation

	Steatosis (+) (n = 54)	Steatosis (-) (n = 49)	P-value
Age (year)	50.92 ± 9.24	51.02 ± 12.02	0.964
Sex (men/women)	35/19	30/19	0.706
Weight (kg)	79.83 ± 16.00	71.89 ± 15.02	0.011
Height (cm)	169.73 ± 9.71	166.35 ± 10.55	0.099
BMI (kg/m^2^)	27.65 ± 5.12	26.11 ± 6.48	0.185
PTDM, n (%)	33 (64.7)	23 (48.9)	0.115
Hyperlipidemia, n (%)	35 (64.8)	24 (49)	0.105
Hypertension, n (%)	40 (74.1)	42 (85.7)	0.143
Etiology, n (%)			0.008
NASH	34 (63)	18 (36.7)	
Non -NASH	20 (37)	31 (63.3)	
AST (IU/L)	26.70 ± 11.88	24.40 ± 12.35	0.352
ALT (IU/L)	34.29 ± 20.53	28.13 ± 20.09	0.142
ALK. Ph (IU/L)	236.33 ± 101.55	258.04 ± 126.44	0.354
T. Bilirubin (mg/dL)	1 ± 4.58	0.93 ± 0.51	0.516
D.Bilirubin (mg/dL)	0.35 ± 0.25	0.33 ± 0.28	0.626
FBS (mg/dL)	148.70 ± 76.35	122.87 ± 54.74	0.059
TG (mg/dL)	234.94 ± 147.76	165.10 ± 81.85	0.006
Cholesterol (mg/dL)	184.94 ± 68.12	190.73 ± 48.48	0.634
HDL (mg/dL)	40.94 ± 12.81	46.15 ± 14.28	0.061
LDL (mg/dL)	94.43 ± 40.54	106.89 ± 33.76	0.105
Rejection, n (%)	19 (36.5)	13 (26.5)	0.280
ARB, n (%)	11 (20.4%)	24 (49%)	0.002

ARB: angiotensin receptor blockers; PTDM: Post transplant diabetes mellitus; BMI: Body mass index; NASH: non-alcoholic steatohepatitis; AST: Aspartate aminotransferase; ALT: Alanine aminotransferase; ALK. Ph: Alkaline phosphatase; FBS: Fasting blood sugar; LDL: Low density lipoprotein; HDL: High density lipoprotein; TG: Triglyceride

**Table 3:** Multivariate logistic regression analysis of risk factors of hepatic steatosis after liver transplantation

	Odds ratio	95% Confidence interval	P-value
Triglyceride	1.005	1- 1.009	0.055
Weight	1.016	0.970–1.054	0.398
Etiology (NASH Vs non-NASH)	0.766	0.245–2.39	0.646
ARB	0.303	0.117–0.784	0.014

ARB: angiotensin receptor blockers; NASH: Non-alcoholic steatohepatitis

**Fig. 1:**
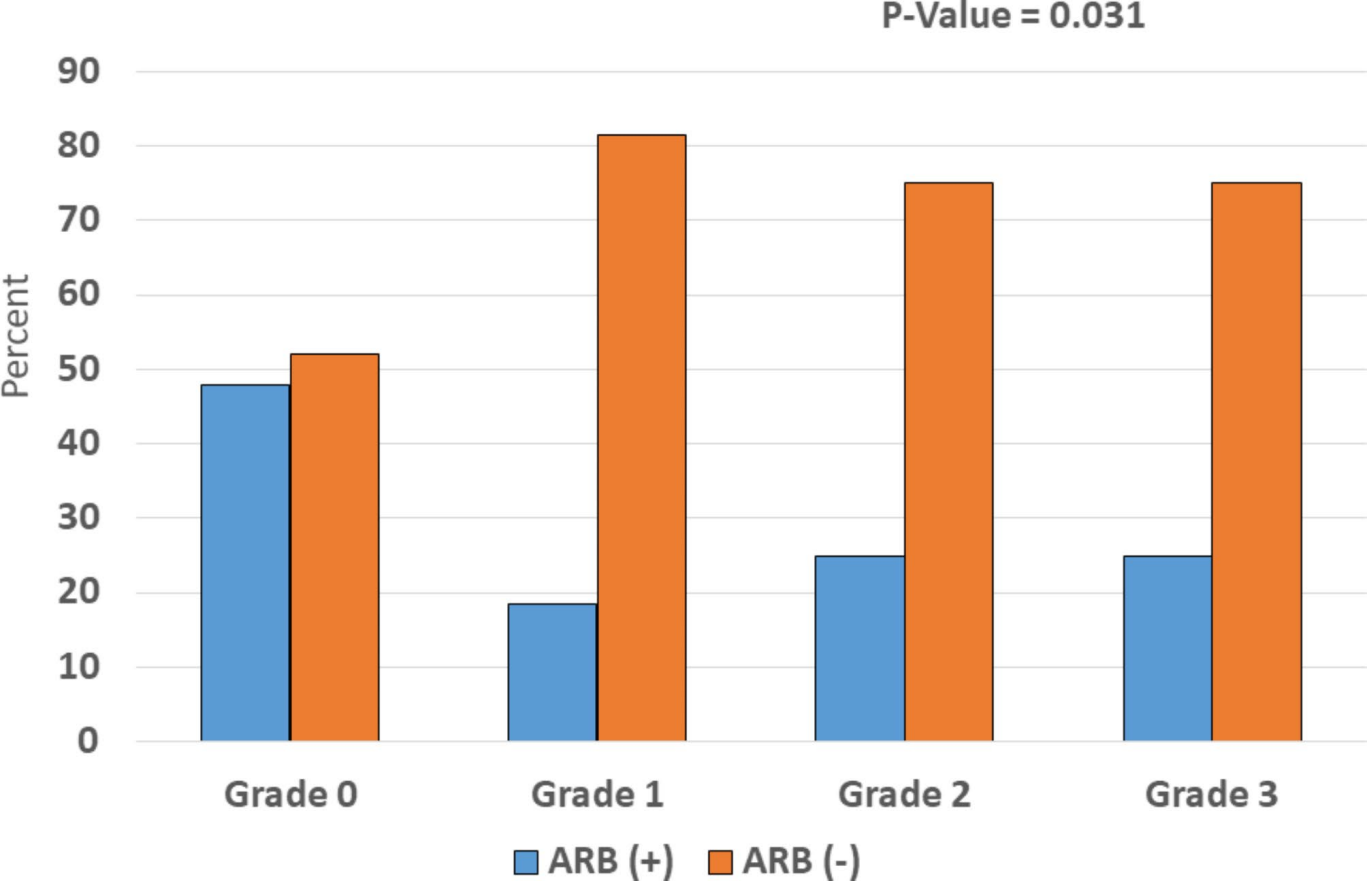
Different grades of hepatic steatosis after liver transplantation in patients with and without ARB treatment

**Fig. 2:**
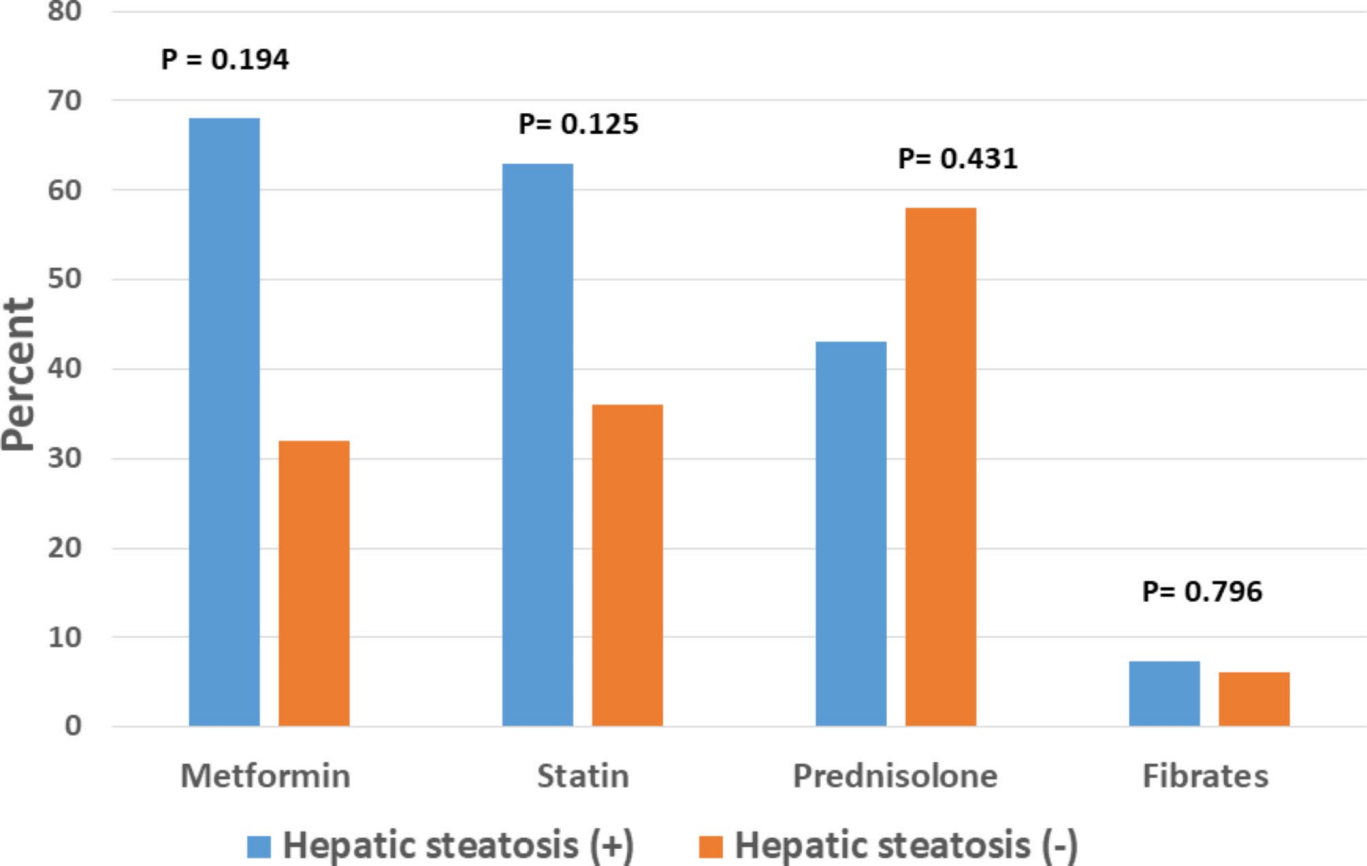
The association between metformin, statins, prednisolone and fibrates use and hepatic steatosis after liver transplantation

Among 35 patients using ARB, 11 patients developed hepatic steatosis. Seven patients had grade 1 hepatic steatosis, 3 patients had grade 2 hepatic steatosis and 1 patient had grade 3 hepatic steatosis. In non ARB users (n = 68), 31 patients had grade 1 hepatic steatosis, 9 patients had grade 2 hepatic steatosis and 3 patients had grade 3 hepatic steatosis after liver transplantation (Fig. 1). Mean duration of ARB use (P = 0.024) and mean cumulative daily dose of ARB (P = 0.015) were significantly lower in patients with hepatic steatosis compared to those without hepatic steatosis after liver transplantation (Table 4). There was no statistically significant difference between losartan and valsartan use in terms of development of hepatic steatosis after liver transplantation (P = 0.722) (Table 4). Liver transplant recipients receiving ARB < 20 months were more likely to develop hepatic steatosis compared to those receiving ARB ≥ 20 months (OR: 0.180; 95% CI: 0.032–0.990 ; P = 0.037). There were no association between metformin use, statin use, prednisolone use and hepatic steatosis after liver transplantation (P > 0.05) (Fig. 2). Comparison of patients with and without hepatic steatosis in a subgroup of patients who used ARB is outlined in Table 5.

**Table 4:** Relationship between ARB use and hepatic steatosis after liver transplantation according to type of ARB, duration and cumulative dose of ARB

	With steatosis	Without steatosis	P-value
Mean duration (months)	23 ± 12.8	38.82 ± 20.11	0.024
Mean cDD (g)	37.77 ± 19. 43	67.98 ± 36.39	0.015
Type of ARB			0.722
Losartan	8 (30. 8%)	19 (69.2%)	
Valsartan	3 (37.5%)	5 (62.5%)	
Duration of use			0.037
< 20 months	5 (62.5%)	3 (23.1%)	
≥ 20 months	6 (37.5%)	20 (76.9%)	
Mean cDDD			0.048
< 20 (g)	4 (66.7%)	21 (75%)	
≥ 20 (g)	7 (25%)	2 (33.3%)	

ARB: angiotensin receptor blockers, cDDD: cumulative defined daily dose; g: gram

**Table 5:** Characteristics of participants with and without hepatic steatosis in a subgroup of patients receiving ARB after liver transplantation

	Steatosis (+) (n = 11)	Steatosis (-) (n = 24)	P-value
Age (year)	49.81 ± 11.89	50.86 ± 11.66	0.809
Weight (kg)	77.09 ± 19.21	70.26 ± 14.64	0.259
BMI (kg/m^2^)	26.16 ± 6.20	25.36 ± 4.65	0.674
PTDM, n (%)	8 (40)	12 (60)	0.314
Hyperlipidemia, n (%)	9 (40.9)	13 (59.1)	0.149
AST (IU/L)	23 ± 6.43	23.19 ± 11.31	0.959
ALT (IU/L)	39.18 ± 12.61	29.14 ± 24.11	0.996
ALK. Ph (IU/L)	209.63 ± 72.09	228.23 ± 105.89	0.606
T. Bilirubin (mg/dL)	1.04 ± 0.57	0.94 ± 0.46	0.601
D.Bilirubin (mg/dL)	0.37 ± 0.27	0.33 ± 0.25	0.712
FBS (mg/dL)	127.90 ± 37.53	126.43 ± 62.40	0.943
TG (mg/dL)	248 ± 144.83	162.09 ± 94.16	0.048
Cholesterol (mg/dL)	184.54 ± 48.65	191.13 ± 55.51	0.740
HDL (mg/dL)	40.09 ± 12.10	40.09 ± 12.40	0.057
LDL (mg/dL)	93.81 ± 31.54	107.81 ± 38.37	0.305
Rejection, n (%)	3 (30)	7 (30)	0.980

ARB: angiotensin receptor blockers; PTDM: Post transplant diabetes mellitus; BMI: Body mass index; AST: Aspartate aminotransferase; ALT: Alanine aminotransferase; ALK. Ph: Alkaline phosphatase; FBS: Fasting blood sugar; LDL: Low density lipoprotein; HDL: High density lipoprotein; TG: Triglyceride

## Discussion

In this study, we found that liver transplant recipients receiving ARB were less likely to develop hepatic steatosis after liver transplantation. Among liver transplant recipients who were treated with ARB, those who received higher defined daily doses of ARB were more protected against hepatic steatosis. Mean cumulative DDD of ARB and duration of ARB were higher in those without hepatic steatosis compared to those with hepatic steatosis after liver transplantation. Our findings showed that use of ARB was safe and well tolerated in post liver transplant period.

Recurrent or de novo hepatic steatosis is highly prevalent after liver transplantation and is increasingly reported in different studies [17, 18]. However, no pharmacologic therapy has been recommended yet. In addition to metabolic abnormalities in post-transplant period [19], several other mechanisms have been proposed for pathogenesis of hepatic steatosis after liver transplantation [20, 21]. Cumulative evidence suggest that renin angiotensin system (RAS) might have crucial role in pathogenesis of NAFLD [22]. The biologically active end–product of RAS is angiotensin II which acts through activation of its two receptors angiotensin receptors I (ATR1) and II (ATR2) [23]. Genetic polymorphisms in ATR1 have been reported to be associated with NAFLD, insulin resistance and post prandial accumulation of cholesterol and triglyceride in the hepatocytes [24, 25]. Wu et al. reported that increased activation of RAS components in high fat diet mice was associated with increased plasma levels of lipid profile [26]. In an animal study on insulin resistant rats, it has been shown that activation of ATR1 contributed to protein oxidation, impaired hepatic lipid and antioxidant metabolism and promotion of hepatic steatosis. In this study, ARB treatment improved lipid profile and metabolic abnormalities [27]. In another animal study, olmesartan, an ARB, treatment was capable of attenuation of hepatic overproduction and accumulation of triglyceride in relation to insulin resistance [28]. Tao et al. showed that angiotensinogen (the precursor of angiotensin peptides) has a main role in hepatic steatosis as hepatocyte angiotensinogen deficient mice have attenuated liver steatosis and less weight gain [29]. Angiotensin II is also a promoter of hepatic fibrosis by activation of hepatic stellate cells (HSC) and up-regulation of fibrosis biomarkers [30]. In another study, it was demonstrated that activation of Janus kinase-2 (JAK-2) induced hepatic fibrosis is mediated through stimulation of ATR1 and prevented by pharmacologic inhibition of JAK-2 [31]. In a rat model of NASH, it has been reported that angiotensin II augmented activation of hepatic stellate cells via toll like receptor-4 signaling and ATR1 [32].

Results of human studies about efficacy of ARB on treatment of NAFLD have been conflicting while promising. In FANTASY trial, no significant improvement in liver enzymes was observed by losartan treatment in patients with NAFLD while telmisartan showed some beneficial effects [33]. In a randomized cross-over trial among pediatric patients with NAFLD, treatment with losartan for 8 weeks was associated with improvement of liver enzymes and insulin resistance compared to the placebo [34]. Combination of losartan and simvastatin was effective in amelioration of liver steatosis and decreasing subcutaneous and visceral adipose tissue in hypertensive patients with NAFLD [35].

This study has several strengths. There has been lack of data about post-transplant treatment of hepatic steatosis. Furthermore, there is no approved pharmacologic therapy for recurrent or de-novo hepatic seatosis after liver transplantation. Our study provided the first clinical evidence suggesting that losartan and valsartan treatment might be protective against hepatic steatosis in liver transplant recipients. We addressed confounding variables by matching each study patient in regard to sex, age, date of liver transplant. We also showed that other medications including statins, metformin and prednisolone had no significant impact on development of hepatic steatosis after liver transplantation. The study is however limited for the retrospective design and rather small sample size. Steatosis was also diagnosed by ultrasound while other diagnostic methods like liver biopsy and controlled attenuation parameter might be better estimates for liver steatosis.

In conclusion, ARB use may have beneficial protective effects against hepatic steatosis in liver transplant recipients. Results of this study, while promising, should be verified in randomized clinical trials before any recommendations for treatment of hepatic steatosis with ARB in this group of patients.

## Data Availability

The datasets generated and/or analysed during the current study are not publicly available due to keeping privacy of patients but are available from the corresponding author on reasonable request.

## Abbreviations

ARB: Angiotensin receptor blockers
PTDM: Post-transplant diabetes mellitus
NAFLD: Non-alcoholic fatty liver disease
NASH: Non-alcoholic steatohepatitis
